# Transcatheter Closure of Patent Ductus Arteriosus via Different Approaches

**DOI:** 10.3389/fcvm.2021.797905

**Published:** 2022-01-10

**Authors:** Zeming Zhou, Yuanrui Gu, Hong Zheng, Shiguo Li, Liang Xu, Qiong Liu, Junyi Wan, Jianhua Lv, Huijun Song, Chaowu Yan, Haibo Hu, Gejun Zhang, Zhongying Xu, Jinglin Jin

**Affiliations:** ^1^State Key Laboratory of Cardiovascular Disease, Fuwai Hospital, Department of Structural Heart Disease, National Center for Cardiovascular Diseases, Chinese Academy of Medical Sciences, Peking Union Medical College, Beijing, China; ^2^State Key Laboratory of Cardiovascular Disease, Fuwai Hospital, Department of Vascular Surgery, National Center for Cardiovascular Diseases, Chinese Academy of Medical Sciences, Peking Union Medical College, Beijing, China

**Keywords:** congenital heart disease, patent ductus arteriosus, interventional, punctures, approach

## Abstract

**Background:** There have been marked advances in devices such as Amplatzer Duct Occluder II (ADO-II) or vascular plug through 5Fr delivery sheath for closure of patent ductus arteriosus (PDA) in the past five decades, making it possible for cardiologists to deliver occluders via different approaches. However, comparisons of these different approaches have not been reported. Therefore, the aim of this study was to summarize and compare the advantages of different approaches for PDA closure, and to guide clinical strategies.

**Methods:** This retrospective study included all patients undergoing transcatheter closure of PDA from 2019 to 2020. Patients were matched by 1:1 propensity score matching (PSM). The retrograde femoral artery approach (FAA) and simple vein approach (SVA) groups were compared with the conventional arteriovenous approach (CAA).

**Results:** The average age of the 476 patients was 21.05 ± 21.15 years. Their average weight was 38.23 ± 24.1 kg and average height was 130.14 ± 34.45 cm. The mean diameter of the PDA was 4.29 ± 2.25 mm. There were 127 men and 349 women, comprising 205 adults and 271 children. Among them, 197 patients underwent CAA, 223 underwent SVA, and 56 underwent retrograde FAA. The diameter in the FAA group was smaller than that in the other two groups, but was similar in adults and children. In the PSM comparison of CAA and SVA, 136 patients with CAA and 136 patients with SVA were recruited. Simple vein approach was associated with markedly reduced length of hospital stay, length of operation, and contrast medium usage as compared with CAA (all *P* < 0.05). In the PSM comparison of FAA and CAA, 30 patients with CAA and 30 patients with FAA were recruited. The operation duration was longer in the CAA than in the FAA group. There were no significant differences in postoperative complications among groups.

**Conclusion:** Patent ductus arteriosus closure by using the SVA and FAA is safe and effective, and has certain advantages in some respects as compared with CAA.

## Introduction

Patent ductus arteriosus (PDA) is one of the most common congenital heart diseases (CHDs), accounting for 5–10% of all CHDs (1, 2). The disease incidence in females is approximately twice that in males. The ductus is an essential tunnel for blood circulation in the fetus, connecting the aorta and the main pulmonary artery. A long-term left-to-right shunt will cause left-side overload and pulmonary artery hypertension. Eisenmenger syndrome manifests as severe pulmonary artery hypertension, with the shunt reversed from left to right. Thus, intervention is recommended if symptoms present.

The first transcatheter closure of PDA was conducted by Porstmann in 1967 (3). To date, transcatheter closure has been the treatment of choice for PDA, particularly given its advantages of minor trauma and a shorter recovery period (4–9). The traditional approach requires puncture of both the femoral artery and vein, which poses the risk of femoral artery aneurysm and femoral arteriovenous fistula. There have been marked advances in devices such as Amplatzer Duct Occluder II (ADO-II) or vascular plug through 5Fr delivery sheath in the past five decades (10–15), making it possible for cardiologists to deliver occluders via different approaches. Currently, there is no study to compare different PDA closure methods. Therefore, the aim of this study was to summarize and compare the advantages of different approaches for PDA closure, and to guide clinical strategies.

## Methods

### Study Population

This study retrospectively examined all patients who underwent transcatheter closure at our institution, the Department of Structural Heart Disease of Fuwai Hospital, between January 1, 2019, and December 31, 2020. This study was carried out in accordance with the recommendations of the Ethics Committee of Fuwai Hospital with written informed consent from all subjects. All subjects gave written informed consent in accordance with the Declaration of Helsinki.

All patients underwent transthoracic echocardiography (TTE), radiography, and electrocardiography before procedure. Patients who successfully underwent interventional therapy were included in the study, except when the PDA closure was combined with other cardiovascular interventional therapies during the same period and when the patient had another CHD requiring surgical repair. The indications for transcatheter closure of PDA were as follows: (1) clinical symptoms associated with cardiac overload performance; (2) bodyweight ≥4 kg; (3) left-to-right shunt across the PDA, (4) an audible cardiac murmur attributed to PDA. Exclusion criteria were endocarditis; resistance pulmonary hypertension, and femoral artery blood oxygen saturation ≤90% (16).

Clinical data were collected from hospital electronic medical records and the picture archiving and communication system. Data on variables such as age, sex, weight, and height were recorded.

### Procedure

#### Simple Venous Approach

In the simple venous approach (SVA), the procedures were performed under local or general anesthesia. The puncture sheath was inserted into the right femoral vein. Heparin (100 IU/kg) was administered after cannulated. A 5 Fr or 6 Fr MPA2 catheter was used in an attempt to cross the PDA directly, or with the assistance of a standard straight wire. The size and shape of the PDA were assessed by TTE, manual pushing of the contrast medium, or pigtail catheter angiography in the PDA or descending aorta. An extra-stiff wire was advanced into the descending aorta and exchanged for the delivery sheath. After the closure device had been implanted, the position of the device and any residual shunt were accessed by TTE and fluoroscopy. Finally, the closure device was released under the guidance of X-ray and TTE.

#### Femoral Artery Approach

In the femoral artery approach (FAA), the procedures were performed under local or general anesthesia. The right femoral artery was punctured, and the puncture sheaths were inserted. Heparin (100 IU/kg) was administered after cannulated. Then, aortography was performed using a 5 Fr pigtail catheter. A 5 Fr delivery sheath was sent into the pulmonary artery directly or under the assistance of an exchange guide-wire. Then, an ADO-II (Abbott, Chicago, IL, USA) or vascular plug (Starway Medical Tec Co., Ltd., Beijing, China) was implanted in the femoral artery. Manual pushing of contrast medium was used to examine the residual shunt and deploy the device. Aortography was repeated, and pressure was measured continuously.

#### Conventional Arteriovenous Approach

In the conventional arteriovenous approach (CAA), the procedures were performed under local anesthesia in adults and under general anesthesia in children (≤10 years old). All patients underwent fluoroscopy-guided procedures. The right femoral artery and vein were punctured, and puncture sheaths were inserted. Heparin (100 IU/kg) was administered after cannulated. Routine right heart catheterization was performed, and a 5 or 6 Fr MPA2 catheter was inserted into the pulmonary artery and right ventricle to measure the pressure. Then, a 5 Fr pigtail catheter was inserted into the descending aorta, and aortography was performed. The shape and diameter of the duct were observed and measured using aortography. The procedure of device deployment was similar to that used in the SVA: a 5 or 6 Fr MPA2 catheter was inserted into the descending aorta, crossing the PDA. This was exchanged for the extra-stiff wire, which in turn, was used to place the delivery sheath.

If the MPA2 catheter could not cross the PDA from the pulmonary artery, the produce was switched to the conventional method: a cut pigtail catheter and a 260-mm 0.035-inch exchange guide-wire was coordinated to pass through the duct, and the exchange wire was sent into the pulmonary artery or superior vena cava. Then, a gooseneck snare was sent through the femoral vein to snare and exteriorize the guide-wire. After the arteriovenous loop was established, the delivery sheath was sent into the aorta via the PDA from the femoral vein. The device was deployed under fluoroscopy and angiographic guidance. Aortography was repeated, and pressure was measured continuously. Finally, the device was released under the guidance of X-ray and TTE, after confirming the correct position.

### Device Detail

The most frequently used device in our institution was the mushroom occluder, produced by Lifetech Scientific Co., Ltd. (Shenzhen, China), Starway Medical Tec Co., Ltd. (Beijing, China), and Shanghai Shape Memory Alloy Co., Ltd. (Shanghai, China). The device and the delivery system were about 1600 US dollars. This type of occluder was similar to the ADO I. The size of the occluder is often 2–4 mm larger than the narrowest diameter of the PDA at the end of the pulmonary artery, or twice the narrowest diameter of PDA.

The ADO-II or vascular plugs are usually used in the long PDA ducts. The ADO-II is braided from 144 nitinol wires, and two symmetrical discs on either side are connected by a middle cylinder, called the waist. The ADO-II device obstructs the blood flow via the compact mesh in the discs on both sides, and has no polyester fabric sewn inside. Thus, the largest ADO-II can be deployed via a 5 Fr sheath. The vascular plug produced by Starway Medical was similar to that produced by ADO-II. However, the middle cylinder had the same length as the disc, and the smallest size had a diameter of 6 mm. Therefore, it is often used as an adjunct to the ADO-II.

### Follow-Up

All patients underwent TTE, X-ray, and ECG on the day after the intervention to detect the position and residual shunt and repeat the examination at 1, 3, 6, 12 month after discharge.

### Statistical Analysis

SPSS 26.0 software (IBM Corp, Armonk, NY, USA) was used for data analysis. Continuous variables are expressed as mean ± standard deviation (SD), and categorical variables as number and percentage. Conventional arteriovenous approach patients were matched to those in the other two groups at a 1:1 ratio using nearest-neighbor propensity score matching (PSM) through R version 3.6.0. Four indices were used in the propensity score model and are presented in **Tables 2**, **3**. Groups were matched without replacement, with a caliper set at 0.02 of a SD of the logit of the propensity score and by using “greedy” matching. Continuous variables were compared using an independent-samples *t*-test or Wilcoxon rank-sum test. Categorical variables were compared using the chi-square test or Fisher's exact test. The correlation between variables was described using linear regression. Statistical significance was set at *P* < 0.05.

## Results

### Population Characteristic

A total of 509 patients were consecutively recruited and underwent transcatheter closure of PDA at the Department of Structural Heart disease of Fuwai Hospital from 2019 to 2020. Twenty-four patients were combined with other cardiovascular interventional therapies. For three patients with small PDA diameters, the catheter coordinated with the guide-wire could not pass through the duct despite numerous attempts. Later angiography showed that the PDA was self-occluded due to thrombosis. In five patients the device was recalled because of severe pulmonary hypertension, and pulmonary artery pressure did not decrease after the occluder was implanted. Device exfoliation occurred in one patient and the operation was performed in time. Finally, 476 patients were recruited for the statistical analysis. All the patients were detected by cardiac dimension enlargement and left-to-right shunt, or an audible cardiac murmur could be heard. Patient characteristics are presented in Table 1. The mean age of all patients was 21 years. Of the patients, 205 were adults and 27.6% were male. Three patients had undergone previous surgical ligation and one patient had undergone previous transcatheter closure, and the residual shunts needed re-intervention.

**Table 1:** Baseline characteristics of patients.

**Characteristic**	***n*** **= 476**
Age (years), mean ± SD	21.05 ± 21.15
BMI	19.44 ± 4.71
Male, *n* (%)	127 (27.6)
Adults, *n* (%)	205 (43.1)
Previous PDA operations	
PDA ligation	3
Transcatheter	1
With other congenital heart diseases	
ASD	6
PFO	18
VSD	2
AS (mild)	10
Mean pulmonary artery pressure, mmHg	23.70 ± 10.36

*PDA, patent ductus arteriosus; ASD, atrial septal defect; PFO, patent foramen oval; VSD, ventricular septal defect; AS, aortic stenosis; BMI, body mass index*.

### Procedural Details

Conventional arteriovenous approach was adopted in 197 patients (41.4%), SVA in 223 patients (46.8%), and FAA in 56 patients (11.8%). An arteriovenous loop was established in 21 patients in the CAA group. In seven of them, SVA was attempted but failed, and the procedure was subsequently switched to CAA. These patients included four adults and three children aged <7 years. Two of the four adults were Type D (Krichenko classification) (17), even if the diameter was >5 mm, while the other two patients were Type A, with a 4-mm diameter. The three patients <7 years of age were classified as Type A with a <3-mm diameter. In the FAA group, ADO-II and vascular plugs were used in 50 and six patients, respectively. In the other two groups, all three types of devices were used.

Length of hospital stay, length of operation time, and contrast medium usage in all CAA cases were greater than those in both the SVA and FAA cases overall. After PSM, age, sex, body mass index, and PDA diameter were similar in the PSM groups. Sixty patients were enrolled in PSM comparison of FAA and CAA, and 272 patients in the PSM comparison of CAA and SVA. The comparison of length of hospital stay, length of operation time, and contrast medium usage in the PSM-SVA group was significantly reduced as compared to the PSM-CAA group, with similar results as found when all CAAs were compared with all SVAs. However, the results for the comparison of the PSM-FAA and PSM-CAA groups were different: length of hospital stay and contrast medium usage were similar, but the operation time was significantly reduced (*P* = 0.036) (Tables 2, 3). We compared PSM-SVA (*n* = 23) and PSM-FAA (*n* = 23). It found that FAA used more contrast medium than SVA (41.09 ± 14.14 vs. 29.70 ± 12.00 ml; *P* = 0.005).

**Table 2:** Summary of baseline of CAA and FAA groups.

	**All-CAA (***n*** = 197)**	**ALL-FAA (***n*** = 56)**	***P*** **-value**	**PSM-CAA (***n*** = 30)**	**PSM-FAA (***n*** = 30)**	***P*** **-value**
Age, years	23.26(0.6–77)	11.29(0.8–51)	0.02	12.23(1.1–57)	12.50(1.3–46)	0.19
Male, *n*	44	25	0.001	8	9	0.77
BMI	19.82 ± 4.84	17.69 ± 3.62	0.003	17.30 ± 3.42	18.22 ± 4.02	0.42
PDA diameter	4.59 ± 2.59	2.10 ± 0.58	<0.001	2.32 ± 0.65	2.29 ± 0.66	0.77
Contrast medium usage (ml)	54.23 ± 27.30	40.09 ± 12.95	<0.001	48.00 ± 22.61	41.33 ± 13.19	0.42
Length of hospital stay (days)	3.96 ± 2.15	3.00 ± 1.16	0.001	3.47 ± 0.26	3.10 ± 0.24	0.31
Length of operation time (min)	39.56 ± 17.05	31.88 ± 9.23	0.001	40.30 ± 15.21	31.70 ± 8.85	0.036

*PDA, patent ductus arteriosus; BMI, body mass index; CAA, conventional arteriovenous approach; FAA, retrograde femoral artery approach; PSM, propensity score matching*.

**Table 3:** Summary of baseline of CAA and SVA groups.

	**All-CAA (***n*** = 197)**	**ALL-SVA (***n*** = 223)**	***P*** **-value**	**PSM-CAA (***n*** = 136)**	**PSM-SVA (***n*** = 136)**	***P*** **-value**
Age, years	23.26 (0.6–77)	21.54 (0.5–77)	0.39	22.26 (0.6–68)	21.69 (0.8–68)	0.89
Male, *n*	44	58	0.38	26	24	0.75
BMI	19.82 ± 4.84	19.54 ± 4.74	0.56	19.99 ± 5.06	19.44 ± 4.86	0.37
PDA diameter	4.59 ± 2.59	4.58 ± 1.85	0.22	4.9 ± 2.38	4.48 ± 1.85	0.36
Contrast medium usage (ml)	54.23 ± 27.30	31.21 ± 16.92	<0.001	52.93 ± 25.38	31.56 ± 16.46	<0.001
Length of hospital stay (days)	3.96 ± 2.15	2.87 ± 1.28	<0.001	3.99 ± 2.23	2.81 ± 1.19	<0.001
Length of operation time (min)	39.57 ± 17.04	28.71 ± 9.86	<0.001	39.61 ± 14.73	29.31 ± 10.72	<0.001

*PDA, patent ductus arteriosus; BMI, body mass index; CAA, conventional arteriovenous; SVA, simple venous approach; PSM, propensity score matching*.

Pulmonary and aortic forward blood flow acceleration was observed in children <7 years old, without obvious obstruction, in one, four, and two CAA, SVA, and FAA patients, respectively, without statistical significance (*P* > 0.05). A minor residual shunt occurred in eight patients, one patient disappeared before discharge, and seven patients disappeared in the follow-up period. In the CAA group, a femoral arteriovenous fistula was found in one patient at the pre-discharge examination, and a femoral aneurysm occurred in three patients. The fistula was approximately 2.0 mm. Local compression was performed for 30 min under ultrasound guidance, and the murmur disappeared, and then a compression bandage was performed. In the FAA group, no femoral aneurysms occurred. No puncture complications occurred in the SVA group. The complications in all of the above cases disappeared during the 1-month follow-up.

### Data Measured by TTE and Angiographic

The morphology of the PDA was observed by angiography. Type A occurred in 55%, type B occurred in 2%, type C occurred in 34%, type D occurred in 6%, and type E occurred in 3%.

The mean diameter of the narrowest PDA was 4.49 ± 2.22 mm (range: 1–14 mm) and 4.29 ± 2.25 mm (range: 1–14 mm), as measured by TTE and angiography, respectively. In children, TTE measurements were statistically significantly larger than angiographic measurements (3.59 vs. 3.34 mm, *P* < 0.001). However, the opposite was found in adults (5.69 vs. 5.55 mm, *P* = 0.17). Although the result in children had a statistical significance (difference = 0.25 mm), and the difference was small and did not affect the clinical strategy, the TTE data correlated with the angiography data (*R*^2^ = 0.70, *P* < 0.001).

The diameters measured by angiography in adults and children in the different groups are shown in Table 4. The diameter of children was significantly smaller than that in adults in all groups (*P* < 0.05), except for FAA (*P* = 0.161), and the diameter of the PDA in the FAA cases was the smallest. In the SVA cases, the diameter in 153 of 223 cases were ≥4 mm, while it was between 2 and 4 mm in 68.

**Table 4:** Diameter of adults and children in different groups.

	**FAA (mm)**	**SVA (mm)**	**CAA (mm)**	**Total (mm)**
Adults (range)	2.33 (1–4)	5.61 (3–14)	5.91 (2–14)	5.55 (1–14)
Children (range)	2.03 (1–3)	3.75 (1–8)	3.40 (1–8)	3.34 (1–8)
*P*-value	0.161	<0.001	0.001	<0.001

*FAA, retrograde femoral artery approach; SVA, simple venous approach; CAA, conventional arteriovenous*.

### Device Application

A total of 381 patients were implanted with mushroom occluders. The mean diameter of PDA was 4.82 ± 2.18 mm (range: 1–14 mm) and the occluder size at the pulmonary end was 9.21 ± 3.84 mm (*R*^2^ = 0.84, *P* < 0.001). Eighty-four patients had ADO-II devices deployed, with a mean PDA diameter of 2.08 ± 0.58 mm (range: 1–4 mm) and mean diameter of the middle cylinder of the device of 4.06 ± 1.06 mm (*R*^2^ = 0.39, *P* < 0.001). In the FAA group, 50 and six patients used ADO-II and vascular plugs, respectively. In the other two groups, all three types of devices were used. In 11 patients, vascular plugs with a diameter of 7.64 ± 1.93 mm were used for PDAs with a mean diameter of 2.91 ± 1.38 mm (range: 1–6 mm).

## Discussion

This study is a short-term study from 2019 to 2020, with no significant change in devices and operator experience during this period. Therefore, after excluding the effect of device and operator, it could be more accurately reflected the difference and advantage of different interventional approaches. In this study, we collected clinical data, including the diameter of the PDA, contrast medium usage, length of hospital stay, and operation time to analyze the advantages of different approaches, in order to share our clinical strategy experience.

### Advantage in SVA

In the CAA approach, the femoral artery and vein were simultaneously punctured, and a pigtail catheter is inserted to perform angiography before and after the device was deployed, to select the appropriate device and to evaluate the situation after device deployment. Femoral arteriovenous fistula, hematoma, pseudoaneurysm, artery occlusion, and other complications are often associated with femoral artery puncture, particularly in children. The artery-related complications mentioned above can be avoided by using SVA. In this study, SVA was used in 100 adult patients and 123 children. Regardless of whether the comparison was performed with or without PSM, contrast medium usage, length of hospital stay, and operation time were significantly reduced when SVA was used rather than CAA.

The PDA diameter was not statistically significantly different between the two groups. In the SVA group, the PDA was evaluated by TTE, manual pushing of contrast medium, or pigtail injection angiography before implantation, and the deployed devices were detected by TTE and/or fluoroscopy. The contrast medium usage was significantly lower in the SVA than in the CAA group. Therefore, SVA could reduce the risk of contrast-induced nephropathy and allergy, particularly in children and older patients with renal insufficiency.

The length of operation time in the SVA group was significantly shorter than that in the CAA group. The operation time in this study was calculated as the time that the patient was wheeled into the cardio-catheter room. Avoiding puncture of the femoral artery could save 5–10 min of press hemostasis. Simultaneously, puncture of the single femoral vein reduces the risk of arterial complications, including artery occlusion and femoral aneurysm. Particularly in children, the unstable strength in pressure hemostasis can explain most occlusions of the femoral artery. The decrease in operation time and contrast medium usage reflected the reduction of radiation, which is beneficial for both doctors and patients (14).

The length of hospital stay in the SVA group was shorter than that in the CAA group due to the 12 h of using a compression bandage in bed, after which patients were discharged.

In general, the SVA group has better results than the CAA group (18, 19). According to our experience, we recommend all PDAs adopt the SVA except for the obstruction of the inferior vena cava or catheter that cannot reach the descending aorta from the pulmonary artery through the PDA. As for children or the elderly with severe calcification, puncturing the femoral artery could be considered (20–22). However, for children, intraoperative TTE was extremely essential to monitor that there is no obstruction of blood flow in the descending aorta especially with large PDA. Conventional arteriovenous approach remains the basic choice for large ducts and those with abnormal morphology (Types B, D, and E).

### ADO II Device and FAA

The ADO-II device can pull back into the 4–5 Fr delivery sheaths, and the sheaths could pass through small-diameter ducts, reaching the pulmonary artery under the support of a guide-wire (15). Thus, these types of devices can be deployed directly via femoral artery puncture sheaths. Amplatzer Duct Occluder II and vascular plugs has replaced coils and become the preferred device to close small-to-moderate PDA (14, 23–25).

After controlling for PDA diameter and other indices, length of hospital stay, and contrast medium usage in the PSM-FAA and PSM-CAA were similar, whereas the length of operation time was reduced in the PSM-FAA group. The reduction in operation time also reflects the improvement of procedures. Compared with the SVA, contrast medium usage was more in the FAA. This may be due to the manual pushing of contrast agent via the sheath before release and/or repeat aortography after release. The diameter of the ADO-II disc was 9–12 mm. Manual pushing of contrast agent combined with TTE sufficed to determine the release condition in adults. Adults with a large descending aorta could avoid repeated angiography, without concerns about obstruction, even when the largest device was implanted. However, children need repeat angiography to monitor for obstruction of the descending aorta. We recommend that the descending aorta diameter should be >10 mm using ADO II (20).

The diameter of the middle cylinder ranged from 3 to 6 mm, with a 1-mm increase between each of the four sizes. In this study, the diameter of most patients ranged from 1 to 3 mm (only one adult had a diameter of 4 mm) and the retrograde FAA in cases with a small PDA achieved better clinical results than in those in whom the CAA was used. The average PDA diameter measured by angiography was larger in adults in the three groups than in children (Table 4). However, the PDA diameters in adults and children in the FAA group were similar. The PDA diameter in the FAA group (2.10 ± 0.58 mm) was significantly smaller than that in patients in whom the other approaches were used. Although, the FAA was adopted in patients with a diameter of <3 mm in our institution, the ADO II device could be used in the diameter up to 5.5 mm (13). And the experience had shown the >3 mm PDA was feasible using Chinese-made occluders and had the same effect as ADO I (23). However, in our institute, the ADO II device was almost twice as the Chinese-made mushroom device (1,400 US dollars vs. 2,700 US dollars). Thus, if the PDA is >3 mm, we usually choose the Chinese-made occluders and adopt the SVA to reduce patients' costs and the risk of artery-related complications.

Femoral artery approach simplified the procedure by avoiding the establishment of arteriovenous loops and the need for multiple attempts or failure to pass through the PDA from the pulmonary artery. In addition, the FAA avoids the concerns about descending aorta obstruction, which requires multiple evaluations prior to release, particularly in children. In children, the descending aorta is slim, and to avoid its obstruction, it is often necessary to manually push the contrast medium via the delivery sheath to observe the shape and position of the occluder before release and to perform continuous pressure measurement and aortic angiography verification after release. If necessary, pulmonary angiography can also be performed by puncturing the femoral vein. According to our institution's experience, the clinical effect of FAA is better than that of CAA. The small, long tube, gourd-shaped, or more tortuous PDA was recommended to adopt the FAA. Especially in the premature low weight infant, the ADO II additional sizes are feasible (26–28).

In recent years, some studies showed that it is feasible to perform PDA occlusion under TTE guidance. It requires the perfect cooperation of the radiologists and the TTE operators and special equipment (29, 30). Choosing a suitable patient is also a key factor. This method may become the first choice in the future.

### Difference in TTE and Angiographic

Transthoracic echocardiography is an indispensable and important method for the preoperative estimation of a PDA. In this study, the measurements obtained by TTE were larger than those obtained by angiography. The color overflowing effect in color Doppler flow imaging causes objects to appear larger than their true diameter (31). Measurements in children obtained by TTE and angiography were statistically significantly different, but those in adults were similar. This phenomenon can be explained by spasms in the ducts stimulated by the catheter and contrast medium, and calcification of the ducts (32). The latter phenomenon is not evident in children. Although the study found that the diameter measured by TTE was larger, it did not affect the clinical strategy. Therefore, TTE was used to estimate the diameter of the PDA and to select an appropriate approach accordingly.

## Limitation

This was a single-center, retrospective study, and the follow-up was incomplete. All specialists who performed operations had over 10 years of interventional closure experience in CHD; thus, real-world success rates of SVA and complications after the operation may be different.

## Conclusion

In summary, with the improvement in proficiency, the number of patients in whom SVA is used is likely to increase, and because of the convenience and safety of new devices, such as the ADO-II, FAA is likely to be used increasingly in small-diameter or long tube-shaped ducts, replacing most of the previous spring-coil embolization methods. Our experience shows that it is safe and effective to adopt SVA and FAA and that these approaches have certain advantages over the CAA.

## Data Availability Statement

The raw data supporting the conclusions of this article will be made available by the authors, without undue reservation.

## Ethics Statement

The studies involving human participants were reviewed and approved by Ethics Committee of Fuwai Hospital. Written informed consent to participate in this study was provided by the participants' legal guardian/next of kin.

## Author Contributions

ZZ and HZ contributed to the design of the study. ZZ and YG contributed to the data collection and analysis, while all authors contributed to data interpretation. ZZ and YG drafted the manuscript and SL, LX, QL, JW, JL, HS, CY, HH, GZ, ZX, and JJ all gave valuable suggestions in the progress of the research. All authors critically revised the manuscript and gave final approval and agreed to be accountable for all aspects of the work, to ensure its integrity and accuracy.

## Funding

This work was supported by National Key Research and Development Program of China (2018YFB1107100).

## Conflict of Interest

The authors declare that the research was conducted in the absence of any commercial or financial relationships that could be construed as a potential conflict of interest.

## Publisher's Note

All claims expressed in this article are solely those of the authors and do not necessarily represent those of their affiliated organizations, or those of the publisher, the editors and the reviewers. Any product that may be evaluated in this article, or claim that may be made by its manufacturer, is not guaranteed or endorsed by the publisher.
